# Enhancement of radiotherapy efficacy by pleiotropic liposomes encapsulated paclitaxel and perfluorotributylamine

**DOI:** 10.1080/10717544.2017.1378939

**Published:** 2017-09-22

**Authors:** Xing Jiang, Baoli Zhang, Zaigang Zhou, Lingtong Meng, Zhiling Sun, Yun Xu, Qiuping Xu, Ahu Yuan, Lixia Yu, Hanqing Qian, Jinhui Wu, Yiqiao Hu, Baorui Liu

**Affiliations:** a The Comprehensive Cancer Centre of Drum Tower Hospital, Clinical College of Traditional Chinese and Western Medicine, Nanjing University of Chinese Medicine, Nanjing, China;; b College of Nursing, Nanjing University of Chinese Medicine, Nanjing, China;; c State Key Laboratory of Pharmaceutical Biotechnology, Medical School of Nanjing University, Nanjing, China;; d The Comprehensive Cancer Centre of Drum Tower Hospital, Medical School of Nanjing University and Clinical Cancer Institute of Nanjing University, Nanjing, China

**Keywords:** Chemoradiotherapy, perfluorotributylamine, paclitaxel, hypoxia, liposomes

## Abstract

Paclitaxel (PTX) is widely used as a radiosensitizer in the clinical treatment of cancer. However, the efficacy of chemoradiotherapy is limited by the hostility of the tumor microenvironment such as hypoxia. To overcome this constraint, we designed pleiotropic radiotherapy sensitized liposomes containing perfluorotributylamine (PFTBA) and PTX. The results showed that liposomes significantly accumulated in the tumor site. PFTBA in liposomes dramatically reversed tumor hypoxia and improved the sensitivity of tumor radiotherapy. PTX in liposomes blocked the cell cycle of tumor cells in the radiation-sensitive G2/M phase, which was even greater when combined with PFTBA. *In vitro* and *in vivo* tumor treatment further demonstrated remarkably improved therapeutic outcomes in radiotherapy with such biocompatible liposomes. In conclusion, the pleiotropic liposomes encapsulated PFTBA and PTX provide significant radiotherapy sensitization and show promise for future application in clinical medicine.

## Introduction

Radiotherapy (RT) is one of the most frequently used treatments for tumors in clinic, and it is reported that more than 50% of cancer patients require RT (Barker et al., 2015). RT uses high-efficiency ionizing radiation to destroy tumor cells by generating free radicals to induce DNA damage (Brown & Wilson, 2004). However, radioresistance occurs in many solid tumors that lead to RT failure. Increasing the dose of RT is not a good option as it could cause serious damage to normal tissue around the tumor. Therefore, improvement in tumor responsiveness to RT is currently a hot spot of clinical research.

The most common radiosensitizers used in current clinical application are chemotherapy, which can enhance DNA damage, inhibit DNA damage repair, or stop the cell cycle in RT sensitive phases. Among these radiosensitizing chemotherapeutic agents, paclitaxel (PTX) has been reported to exhibit radioenhancement in several cancers by blocking the tumor cell cycle in G2/M phase (Safran et al., 1999; Lapidus et al., 2004; Hiro et al., 2010; Xu et al., 2017). However, the effects of chemoradiotherapy were severely limited by the hostility of the tumor microenvironment, and hypoxia was the most formidable problem.

Hypoxia-associate resistance often occurs in RT and chemotherapy of solid tumors, and the degree of hypoxia in tumors seriously affects the survival rate of patients (Mehlen & Puisieux, 2006). Due to its irregular angiogenesis and cell proliferation, the tumor microenvironment is more hypoxic than normal tissue (Vordermark & Horsman, 2016). In RT, ionizing radiation generates DNA radicals that are fixed by O_2_ in normoxic tissues causing DNA damage and cell death. The degree of cellular damage caused by ionizing radiation is directly related to the level of cellular oxygenation. In other words, hypoxic tumor environment can reduce the RT effect, causing radiation resistance. In addition to direct effects of low oxygen on chemoradiosensitivity, hypoxia up-regulates the expression of hypoxia-inducible factor-1 (HIF-1) which promotes resistance through several HIF-1-mediated genes and signaling pathways then dramatically contributes to the aggressiveness and resistance of multitudinous tumors (Karakashev & Reginato, 2015). For instance, hypoxia/HIF-1 upregulates several multidrug resistance genes that reduce the intracellular concentrations of chemotherapeutic drugs by increasing the drug outflow from cancer cells thereby reduce chemoradiotherapy efficacy (Kruh & Belinsky, 2003). Hypoxia was also found to inhibit apoptosis in PTX treated breast cancer cell lines through activation of the c-Jun *N*-terminal kinase pathway (Notte et al., 2013). The hypoxia/HIF-1 effects inhibit apoptosis and promote tumor cell survival in chemoradiotherapy by inhibition of proapoptotic and induction of anti-apoptotic genes.

Several methods have been developed to sensitize hypoxic tumor cells in chemoradiotherapy by supply oxygen to solid tumor, such as modified hemoglobin (Murayama et al., 2012), intravenous perfluorochemicals (Song et al., 2016, 2017; Xu et al., 2016), oxygen-producing drugs (Zhang et al., 2017), as well as other methods. Perfluorochemicals, such as perfluorotributylamine (PFTBA) offers a promising way to increase oxygen levels within tumor tissues. Perfluorochemicals have been widely used as a successful oxygen carrier due to their chemical and biological inertness, ease of sterilization and especially their reliable biosecurity and high solubility for oxygen which is 5–10-fold greater than plasma (Sharts et al., 1978; Lowe et al., 1998; Riess, 2005; Castro & Briceno, 2010). Fluosol-43 (PFTBA emulsion) is approved by the FDA to improve myocardial oxygenation (Young et al., 1990; von der Hardt et al., 2004; Maevsky et al., 2005; Castro & Briceno, 2010). PFTBA has high oxygen solubility and releases oxygen by simple diffusion via an oxygen concentration gradient, which shows excellent RT sensitization potential. Drug delivery systems are needed that will ensure oxygen concentration gradient-dependent release does not occur in blood vessels until it reaches and can accumulate at the tumor site.

Nanotechnology offers an alternative strategy to present drugs to the tumor locations through enhanced permeability and retention (EPR) effects and targeted strategies (Dubey et al., 2012; Tang et al., 2016; Zhang et al., 2016). In addition to the protection and directional delivery of PFTBA, the use of nanotechnology can also reduce the serious toxicity of normal tissue caused by the nonspecific distribution of chemotherapy, especially when combined with RT, which always limiting the clinical application of chemoradiotherapy (Yu et al., 2017).

Therefore, simpler and more efficient nanoagent strategies are needed to overcome tumor hypoxia and enhance tumor chemoradiotherapy. In our previous study, perfluorochemicals loaded nanoparticles have been proven to increase the efficacy of photodynamic therapy by supplying oxygen to the tumor site (Ren et al., 2017). We believed that PFTBA could be used as an effective way to reverse the tumor hypoxia and enhance the efficacy of RT. The classic chemotherapeutic agent PTX is often used as a RT sensitizer, but the therapeutic effects on many types of tumors were limited by tumor microenvironment such as hypoxia. Therefore, in our study, we integrated the advantages of both PFTBA and PTX, and designed pleiotropic liposomes with core–shell structures containing both PFTBA and PTX to increase tumor radiosensitivity. Liposomes can increase the accumulation of drugs in tumor tissues. PTX can induce cell cycle arrest of tumor cells in radiosensitive phases, and PFTBA increases oxygen concentrations at tumor sites that improve radiation-induced tumor damage. All the components in our liposomes are safe and could be used in clinical in future. With great synergistic therapeutic outcome during the combined RT *in vivo*, as well as the no noticeable systemic toxicity after treatment, our pleiotropic liposomes might be a promising radiosensitizer.

## Materials and methods

### Materials, cells and animals

Lecithin was purchased from Aladin Industrial Corporation, DSPE-PEG2000 was purchased from A.V.T. Pharm. Ltd. (Shanghai, China), PFTBA was purchased from Bailingwei Tech Co. Ltd. (Beijing, China), Paclitaxel (PTX) was purchased from Hongdoushan Co. Ltd (Jiangsu, China), and IR775 was purchased from Sigma-Aldrich Chemical Corporation (St. Louis, MO, USA).

Mice colon cancer cells (CT26 cells) were cultured in 25 cm^2^ glass culture flasks dissolved in 5 ml RPMI 1640 supplemented with 10% (v/v) fetal bovine serum (FBS), 100 IU/ml of penicillin G sodium, and 100 mg/ml of streptomycin sulfate. Cells were incubated at 37 °C with 5% CO_2_/95% humidified air, and then sub-cultured three times per week. Cells were harvested in the exponential growth phase for all experiments.

Animals were bought from the Medical Center of Yangzhou University (Yangzhou, Jiangsu, China). Male Balb/c mice (∼20 g) were used to establish an allograft tumor model. Briefly, 1 × 10^7^ CT26 cells in 0.2 ml PBS were subcutaneously injected into right flank of mice. Ten days later, tumors were isolated and cut into small blocks (∼1 mm^3^). Tumor blocks were then implanted into the right flank of healthy mice to develop the tumor model. All animal studies were performed in compliance with guidelines set by the Animal Care Committee at Drum Tower Hospital, Nanjing, China.

Radiotherapy was carried out using a linear accelerator source (Clinac MH-J-16-I, China) at room temperature.

### Preparation and characterization of liposomes

Liposomes were prepared by ultrasonication according to our previously reported study. Briefly, liposome colloidal suspensions were prepared by dissolving 30 mg DSPE-PEG2000 and 120 mg lecithin. Then, 0.33 ml of PFTBA and 200 µl of a 20 mg/mL PTX ethanol solution were gradually added to the suspensions during ultrasonication at 325 w/min for 6 min in an ice bath to form 3 ml lip(PFTBA + PTX). The control groups of lip(PFTBA) and lip(PTX) were formulated respectively as described above. Drug encapsulation efficiency (EE) and drug loading capacity (LC) of PTX in lip(PFTBA + PTX) were investigated by high-performance liquid chromatography (HPLC) with a Zorbax C18 column (150 × 4.6 mm, 5 µm; Agilent Technologies, Santa Clara, CA, USA). The mobile phases included a mixture of acetonitrile and water (52/48 v/v). The injection volume was 20 µL and the wavelength was set at 227 nm. The column temperature was 30 °C. EE and LC were calculated as follows: EE%= (weight of the drug in liposomes/weight of the feeding drug) × 100%, LC%= (weight of the drug in liposomes/total amount of liposomes) × 100%. Drug release was studied in a release media (PBS containing 2% fetal bovine serum) using a dialysis bag with a molecular weight cut off of 10 kDa at 37 °C for 96 h. The PTX content was detected using HPLC. For uptake *in vitro* and to track *in vivo*, IR775, a near infrared (NIR) dye, was added to the liposome colloidal suspensions to form lip(IR775). The morphology of lip(PFTBA + PTX) was characterized by transmission electron microscopy (TEM, JEM-2100 Japan). Particle size distribution of liposomes was detected by dynamic light scattering (DLS, 90Plus, Brookhaven Instrum. Corp). To evaluate the stability of lip(PFTBA + PTX), liposomes were kept at 25 °C and the diameter determined by DLS for 72 h. The concentration of oxygen in different solutions of normal saline (NS), lip(PTX), lip(PFTBA) and lip(PFTBA + PTX) were measured using Clark oxygen electrode.

## Cell experiments

### Cellular uptake *in vitro*

To evaluate the uptake of liposomes in tumor cells *in vitro*, CT26 cells were seeded at a density of 2 × 10^4^/well into glass bottom dishes (Sunnyvale, CA, USA). After 24 h, media was replaced by media containing lip(IR775), or free IR775. After 2 h incubation, cells were washed twice with PBS and fixed with 4% paraformaldehyde solution for 20 min, washed with PBS, and then stained with DAPI for an additional 15 min. Fixed cells were observed by confocal laser scanning microscopy (Olympus FV1000). The excitation/emission wavelengths for IR775 were 774/792 nm.

### Cell-cycle analysis

To assess the influence of NS, lip(PTX), lip(PFTBA) and lip(PFTBA + PTX) on the cell cycle, a propidium iodide (PI)/RNase buffer staining kit (BD Pharmingen, San Jose, CA, USA) was used according to manufacturer’s instructions. 1 × 10^6^ CT26 cells were plated into 6-well plates. After cells attached to the wells, they were treated with NS, lip(PFTBA), lip(PTX), lip(PFTBA + PTX). Cells were collected 24 h later, washed with cold phosphate-buffered saline twice, fixed in 75% ethanol at −20 °C for at least 2 h, washed with cold phosphate-buffered saline, stained with PI/RNase staining buffer for 15 min, and then measured using flow cytometry.

### Clonogenic survival assay *in vitro*

Radiosensitivity was determined using a clonogenic survival assay. CT26 cells (100–800 per well) were plated into six-well plates. After cells attached to the wells, cells were treated with NS, lip(PFTBA), lip(PTX), lip(PFTBA + PTX)(PFTBA 1.25%V/V, PTX 0.167 mg/ml) for 24 h and the RT groups were exposed to increasing doses of IR (0, 2, 4, 6, and 8 Gy) additionally. After 7–10 days of incubation, colonies composed of greater than 50 cells were observed. Surviving fractions (SFs) were calculated as indicated: SF = (the number of colonies formed after treatment/the total number of colonies formed) × 100%.

### Apoptosis assay *in vitro*

Apoptosis assays were performed using the PI staining kit according to manufacturer’s instructions. The non-RT groups were incubated with NS/different liposomes and then incubated for 48 h after treatments. The RT groups were incubated with NS/different liposomes for 24 h, and then received RT, followed up by another 24 h incubation. After incubations, 1 × 10^6^ CT26 cells were collected and washed with PBS. Cells were washed and resuspended in the PI labeling solution (100 μL of 4-(2-hydroxyethyl)-1-piperazineethanesulfonic acid buffer, and 5 μL of PI), and incubated in the dark for 5 min at room temperature. CT26 apoptosis was analyzed using flow cytometry.

## Animal experiments

### Behavior *in vivo*

To study the distribution and accumulation of liposomes in tumor bearing Balb/c mice, IR775, an NIR dye, was loaded into liposomes to form lip(IR775) and was used for *in vivo* fluorescence imaging, while free-IR775 was used as a control. CT26 tumor bearing balb/c mice were injected with lip(IR775) or free-IR775 via the tail vein, and then received whole body optical imaging for 0–72 h using the CRI maestro system that detects NIR fluorescent signals *in vivo*. Exposure time was set to 1 s. Anesthetized mice were placed on animal plates and were heated to 37 °C. Images were analyzed using IVIS Living Imaging Software. To further track the distribution of lip(IR775), different organs including heart, liver, spleen, lung, kidney, intestine, and tumor were harvested and imaged 72 h after administration.

To assess the accumulation behavior of lip(PFTBA + PTX) in tumor directly, Balb/c mice with 200 mm^3^ CT26 tumors in the armpits of right anterior were randomly divided into five groups (three mice per group) and intravenously administrated with lip(PFTBA + PTX) at a dose of 13 mg/kg PTX, respectively. At time points (2, 12, 24, 48 and 72 h) following administration, three mice of each group were sacrificed with the tumor collected then weighted and grinded with morcellator. Homogenate in 3 mL water was extracted with ethyl acetate. The mixture was vortexed for 3 min and centrifuged at 6000 rpm for 10 min. The supernatant was blown dry by nitrogen, and dissolve again by 100 µL acetonitrile. Then, PTX concentration in these samples were measured by HPLC.

### Antitumor efficacy and toxicity evaluation *in vivo*

To evaluate the hypoxia evolution post various treatments, Balb/c mice bearing CT26 tumors were intravenously injected with 0.2 ml NS, lip(PFTBA), lip(PTX) or lip(PFTBA + PTX). Twenty-four hours after the injections, tumor tissues were collected to identify HIF-1α expression using immunohistochemistry.

To assess antitumor efficacy, tumor bearing mice were randomly divided into eight groups, where each of the original groups were divided into receiving RT or not (NS ± RT, lip(PFTBA)±RT, lip(PTX)±RT and lip(PFTBA + PTX)±RT). Each group received intravenously injections on day 0 and 3, and each RT group received subsequent RT 24 h postadministration. For RT, mice were anesthetized with 2% pentobarbital sodium, and tumors were exposed to local radiation at a dose rate of 5 Gy. The region outside the tumor was shielded.

Tumors size and mice weight were measured every day for 14 days after treatment. For treatment evaluation, tumor volumes were calculated using the formula: volume = width^2^×length/2. In order to reduce the effect of initial tumor volume differences among the different groups, relative tumor volumes were calculated using the following formula: [tumor volume on day t (*V*_t_)/the tumor volume on day 0 (*V*_0_)] × 100%. After finished the experiment, major organs were harvested including heart, liver, spleen, lung, and kidney from each group. Organs were fixed in 10% formalin, processed routinely into paraffin sections and stained with hematoxylin and eosin (H&E), then examined by a microscope to estimate systemic toxicity.

To evaluate the therapeutic effect of each treatment group, one mouse from each group was sacrificed three days after treatment. Tumors were collected and subjected to the following methods: H&E histological staining, TUNEL staining, and staining for γ-H2AX expression. Briefly, tumor tissues were harvested and fixed in 10% formalin. Next paraffin-embedded 4 mm thick sections were cutted for histological staining, immunohistochemistry, and immunofluorescence. The paraffin slices were observed by fluorescence microscopy (Nikon, Tokyo, Japan).

### Statistical analysis

The data are presented as mean ± SD, and comparison of mean values was performed using the Student’s *t*-test or ANOVA. A *p* < .05 was accepted as a statistically significant difference.

## Results and discussion

### Preparation and characterization of liposomes

**Figure 1.**
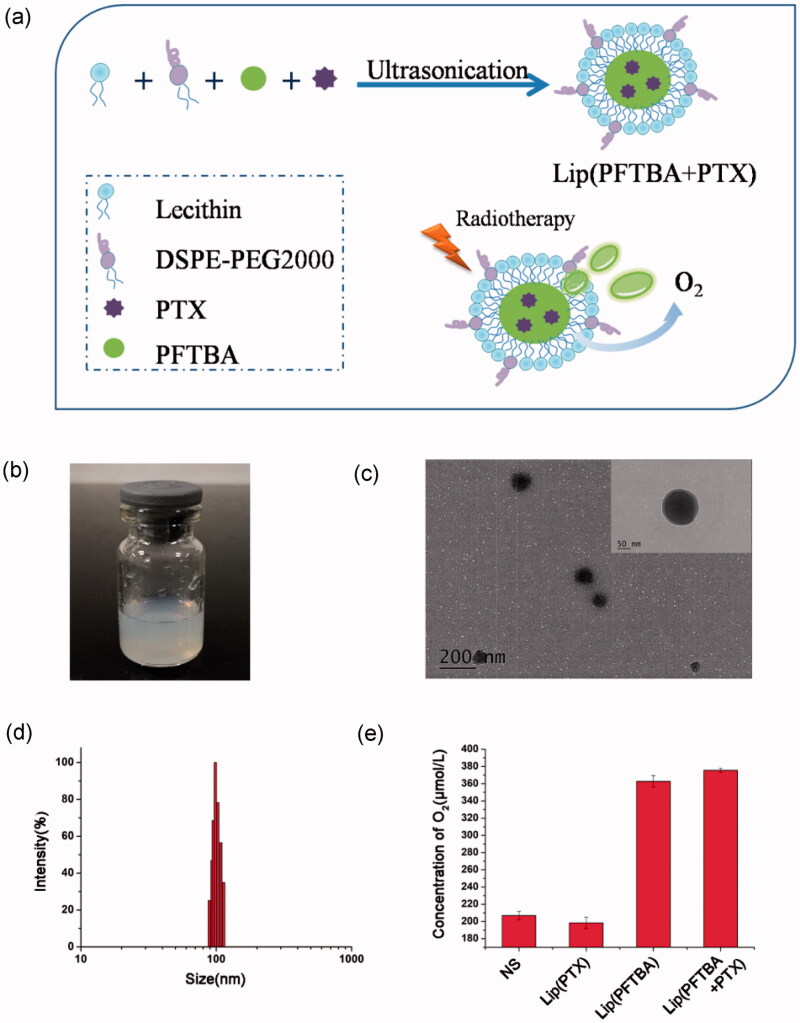
Preparation and characterization of liposomes. (a) Schematic of preparation of lip(PFTBA + PTX). (b) Photograph showing of lip(PFTBA + PTX). (c) Transmission electron microscopy visualization of lip(PFTBA + PTX). (d) Dynamic light scattering measurement of lip(PFTBA + PTX). (e) The O_2_ concentration in different solutions of NS, lip(PTX), lip(PFTBA) and lip(PFTBA + PTX).

We have developed a one-step method to prepare lip(PFTBA + PTX) (Figure 1(a,b)). As shown in the transmission electron microscopy (TEM) image (Figure 1(c)), liposomes were uniform in size. Moreover, the TEM magnification revealed a shell–core structure (inset of Figure 1(c)). Using dynamic light scattering, the mean liposome size was about 100 nm (Figure 1(d)), which is consistent with the TEM observations. No apparent changes in the size of the lip(PFTBA + PTX) were noticed within 72 h which showed nice stability *in vitro* (Figure S1). The size of lip(PFTBA + PTX) is small enough to avoid clearance by the reticuloendothelial system (RES) and to permeate tumor tissue due to its EPR effects. Besides the nanosize, the adoption of DSPE-PEG2000 as a “stealth ghost” on the surface of lip(PFTBA + PTX) could extend resident time *in vivo* (Peer et al., 2007; Davis et al., 2008), which further contributes to the great tumor accumulation demonstrated in subsequent experiments. EE and LC of PTX in lip(PFTBA + PTX) were 88.5 ± 3.8% and 2.3 ± 0.1%. The *in vitro* release behavior was investigated as shown in Figure S2. The releasing of PTX from lip(PFTBA + PTX) were kept for 96 h, with specific releasing rates of 15% and 68% at 24 h and 96 h, respectively. Therefore, lip(PFTBA + PTX) presented a perfect slow-releasing performance. According to the above method, we also prepared lip(PFTBA) and lip(PTX) for comparison in next experiments. In order to detect the effect of PFTBA on the oxygen content of the formulation, we tested the oxygen concentrations in different solutions using the Clark oxygen electrode. Results showed that the oxygen concentrations in the formulations of lip(PFTBA + PTX) and lip(PFTBA) were significantly increased compared to NS and lip(PTX) (Figure 1(e)).

### Cellular uptake *in vitro*

**Figure 2.**
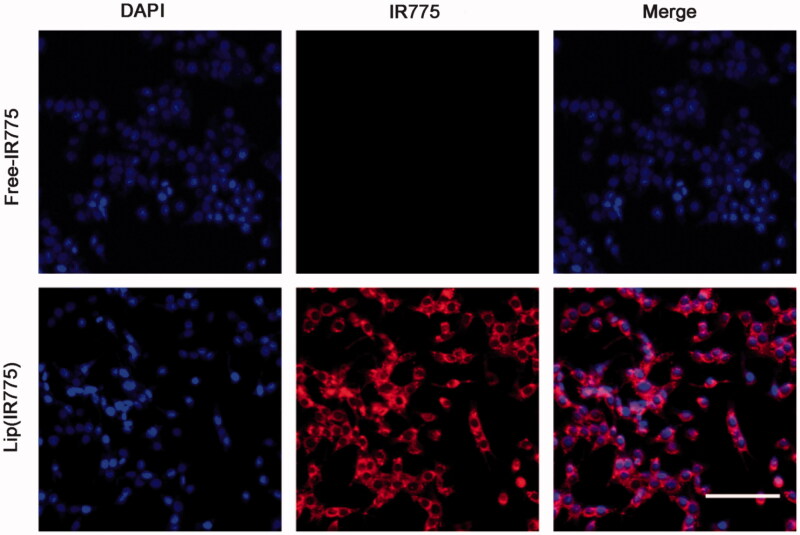
Cellular uptake of liposomes *in vitro*. Confocal laser-scanning microscopy of CT26 cells after 2 h co-incubation with lip(IR775) or free-IR775. IR775 was red and the cancer-cell nucleus were stained blue with DAPI (bar =50 μm).

Cellular uptake of liposomes *in vitro* was confirmed by laser-scanning confocal microscopy after incubation of tumor cells with lip(IR775) for 2 h. It can be seen in the fluorescence images that red punctuate (IR775) points gathered around the nucleus (visualized with blue DAPI dye), and overlapped each other (Figure 2). Compared to lip(IR775), the control group of free IR775 showed no IR775 signal in the cell, meaning that the liposomes could enhance the cellular uptake of drugs.

### Radiosensitization *in vitro*

**Figure 3.**
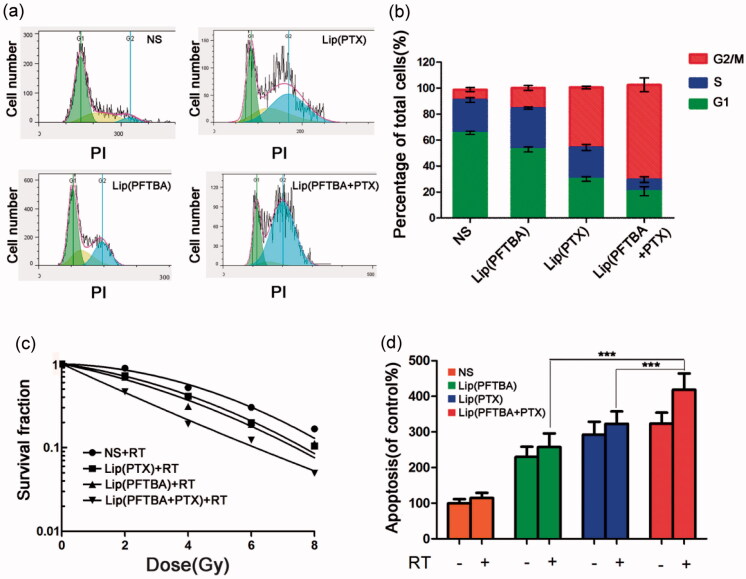
Radiosensitization *in vitro*. (a, b) Effect of NS, lip(PTX), lip(PFTBA) and lip(PFTBA + PTX) on cell-cycle in CT26 cells for 24 h. (c) Clonogenic survival assay carried out after eight days of incubation. (d) Cell apoptosis detected by PI staining at 48 h after being exposed to various conditions. Data are shown as mean ± SD (*n* = 3, ***p < .001).

We next determined the radiation enhancement ability of liposomes *in vitro*. CT26 cells were cultured under a low oxygen environment (1% oxygen). Firstly, we studied the effect of PFTBA or PTX on the tumor cell cycle. Compared to the control group, treatment with lip(PTX) and lip(PFTBA + PTX) induced cell cycle arrest at the G2/M phase markedly. Among them, the effect of lip(PFTBA + PTX) was more significant (Figure 3(a,b)). According to previous reports, cells arrested in the G2/M phase are more sensitive to the damaging effects of radiation (Cui et al., 2014). Therefore, clonogenic survival assays were carried out to clarify the influence of PFTBA and PTX on RT efficacy. As illustrated in Figure 3(c), compared with the NS + RT group, both lip(PTX) and lip(PFTBA) sensitized CT26 cells to the effects of RT, and lip(PFTBA + PTX)+RT significantly decreased the survival rate of CT26 cells compared to the other groups receiving RT. Theses *in vitro* experiments demonstrate that lip(PFTBA + PTX) is a good radiosensitizer.

We further performed flow cytometry to identify the effect each group had on tumor cell apoptosis. As shown in Figure 3(d), cellular incorporation of lip(PFTBA), lip(PTX) and lip(PFTBA + PTX) that received RT increased cell apoptosis compared with control groups that did not receive RT. Nevertheless, the lip(PFTBA + PTX) group was the only group to show pronounced apoptotic rates when combined with RT.

Through these experiments *in vitro*, we found that lip(PTX) alone was able to arrest the cell cycle in the G2/M phase, but not as well as lip(PFTBA + PTX), and that the effect of lip(PFTBA) on RT sensitization was minor either compared that of lip(PFTBA + PTX). In other words, only liposomes that contained the two drugs, lip(PFTBA + PTX), could induce striking cell cycle arrest and show marked tumor cell sensitization to RT, *in vitro*.

### Behavior *in vivo*

**Figure 4.**
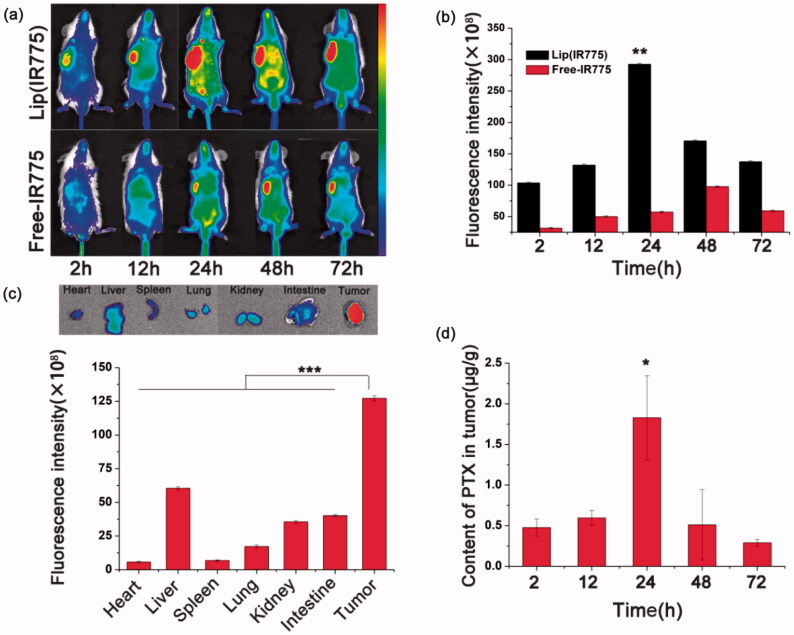
Behavior *in vivo*. (a) *In vivo* dynamic fluorescence imaging after intravenous injection of lip(IR775) or free-IR775. (b) Fluorescence intensity in tumor site by IVIS Living Imaging Software (**p < .01). (c) *Ex vivo* fluorescence images and fluorescence intensity of major organs and tumor of lip(IR775) group at 72 h postinjection (***p < .001). (d) Quantitative analysis of PTX in tumors after intravenous injection of lip(PFTBA + PTX) (*p < .05).

Fluorescence images were acquired on the CRI maestro system at different time points after intravenous injection of lip(IR775) and free-IR775. As shown in Figure 4(a,b), the fluorescence signals from the tumor region of the lip(IR775) group strengthened with time and reached maximum at 24 h, showing a remarkable accumulation of IR775 in the tumors compared to those of the free-IR775 group. The tumor and major organs of the lip(IR775) group were harvested and imaged *ex vivo* 72 h after administration. As shown in Figure 4(c), most liposomes accumulated in the tumor, and the fluorescence signals were greater in the tumor than in the other organs. The preferential lip(IR775) accumulation in tumors might be due to the EPR effects of liposomes. These results showed that by 72 h post-injection, a large concentration of liposomes is found at the tumor site. To more directly quantitative analysis the accumulation behavior of lip(PFTBA + PTX) within the tumor, we analyzed the PTX content in tumor tissues. Results showed that PTX gradually accumulated within the tumor after intravenous injection, reaching the maximum accumulation at 24 h (Figure 4(d)). Then, it retained in the tumor site for a long time even 72 h after drug given. Together, our study results indicate that liposomes can achieve high accumulation in tumors and can remain within tumors for extended periods. Since concentrations of liposomes were highest at 24 h compared to the other time points, RT was performed 24 h after intravenous liposomes injections in subsequent *in vivo* antitumor assays.

### Antitumor efficacy and toxicity evaluation *in vivo*

**Figure 5.**
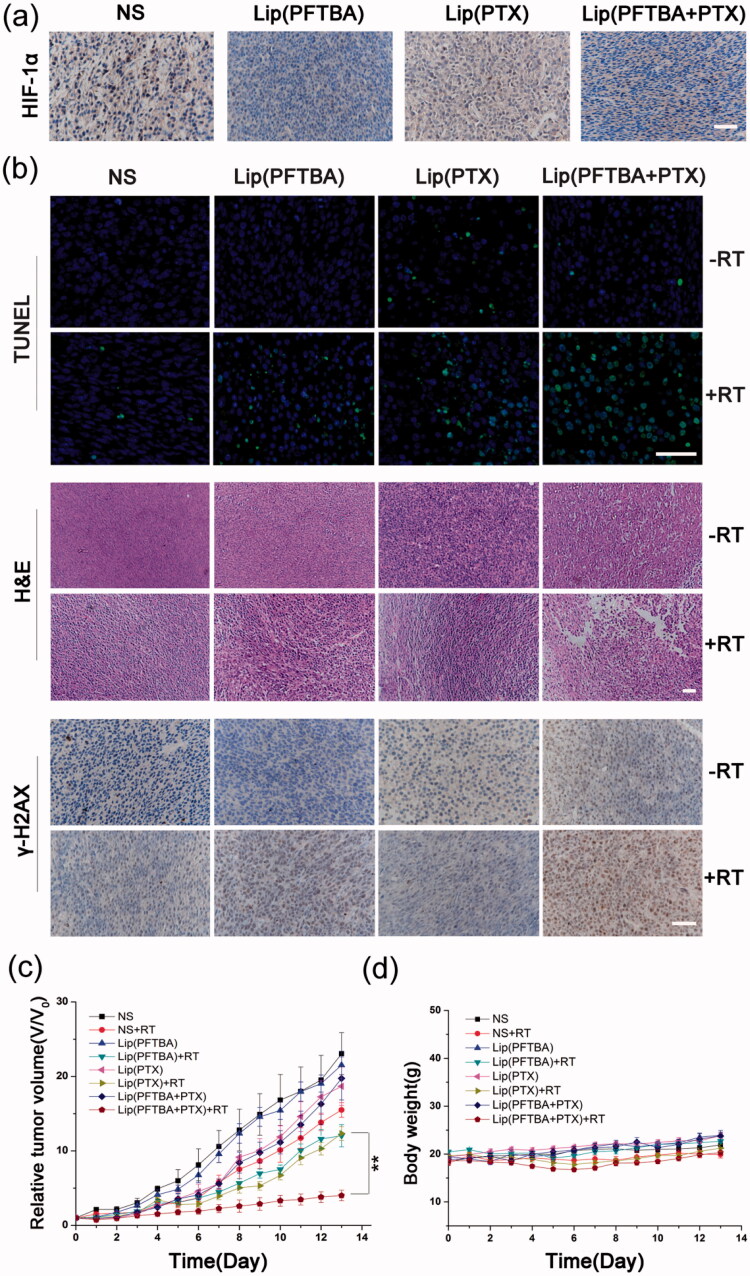
Antitumor efficacy *in vivo*. (a) HIF-1α in tumor tissue identified by immunohistochemical analysis after intravenous injection (staining of HIF-1α, brown; nucleus of cancer cells, blue) (bar =50 μm). (b) TUNEL staining for apoptosis in tumor sections. DAPI counterstaining indicates the tumor nuclear region (bar = 50 μm). H&E staining for pathological changes in tumor sections (bar =50 μm). γ-H2AX staining for double-strand DNA breaks (staining of γ-H2AX, brown; nucleus of cancer cells, blue) (bar =50 μm). (c) Relative tumor volume of different groups of mice after various treatment. Data are expressed as mean ± SD (*n* = 5 mice per group, **p < .01). (d) Changes of body weight of mice in different groups during treatments. Data are expressed as mean ± SD (*n* = 5 mice per group).

To observe the effect of each group on local hypoxia of the tumor, we examined HIF-1α expression in tumor tissues after intervention. HIF-1, as the key molecular signature for hypoxia, is the main downstream regulator of the hypoxic responses in tumor cells (Semenza, 2012). HIF-1 is a transcription factor that is a heterodimer containing two subunits: the hypoxia inducible factor 1 alpha (HIF-1α) and the hypoxia inducible factor 1 beta (HIF-1β). Under normal oxygen concentrations, HIF-1α is rapidly degraded (Semenza, 2000). In our experiments, we investigated the effects on hypoxia/HIF-1α of different groups of liposomes in tumor tissues. After intravenous injection of NS, lip(PFTBA), lip(PTX) and lip(PFTBA + PTX) for 24 h, HIF-1α in the tumor tissue was identified using immunohistochemistry. As shown in Figure 5(a), only lip(PFTBA) and lip(PFTBA + PTX) significantly reduced the HIF-1α expression in tumor tissue compared with NS and lip(PTX), which suggests that the formulation containing PFTBA could reverse intratumor hypoxia.

Next, to evaluate treatment efficacy regarding tumor cell death, TUNEL staining of tissue sections from the different treatment groups was performed 72 h after intravenous injections with or without RT. A large number of apoptotic cells on histological sections were seen in the lip(PFTBA + PTX) group with RT at a dose of 5 Gy when compared with the other groups (Figure 5(b)). H&E staining of tumor sections was also performed coming with TUNEL (Figure 5(b)). Compared with no RT, all RT groups showed tumor necrosis, especially in the lip(PFTBA + PTX)+RT group. These results demonstrated successful tumor cell destruction by the lip(PFTBA + PTX)+RT group.

To investigate the mechanism of the marked synergistic effects associated with lip(PFTBA + PTX) and RT, γ-H2AX, one of the most sensitive indicators of double-strand DNA breaks (Kinner et al., 2008), was evaluated for double-strand DNA damage caused by each group using immunofluorescent labeling (Figure 5(b)). Consistent with the above cell viability and tissue section staining results, lip(PFTBA + PTX)+RT resulted in the highest level of double-strand DNA damage among all groups. The result clearly shows excellent radiosensitization by lip(PFTBA + PTX).

The antitumor effects were monitored by measuring tumor volumes for 14 days. The mice in eight random groups were intravenous injected when a tumor reached ∼60 mm^3^. After 24 h, mice in the RT groups were irradiated with 5 Gy RT additionally. The tumor sizes were normalized to their initial sizes. As shown in Figure 5(c), the anti-tumor effects of the RT groups were better than that of corresponding non-RT groups, and lip(PFTBA + PTX)+RT group showed a remarkable delay in tumor growth compared to that of the lip(PTX)+RT and lip(PFTBA)+RT groups (*p* < 0.01).

**Figure 6.**
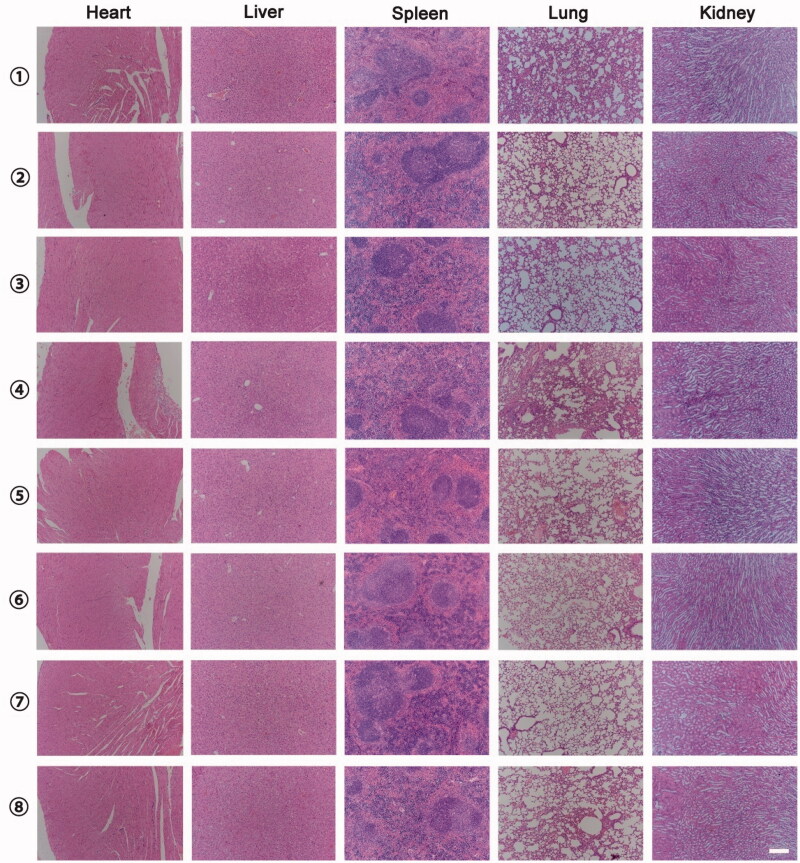
Representative H&E-stained slices images of major organs in each group at the end of the experiment (①NS; ②Lip(PFTBA); ③Lip(PTX); ④Lip(PFTBA + PTX); ⑤NS + RT; ⑥Lip(PFTBA)+RT; ⑦Lip(PTX)+RT; ⑧Lip(PFTBA + PTX)+RT (bar =200 μm).

The body weight of animals was recorded every day, indicating that lip(PFTBA + PTX) was not toxic (Figure 5(d)). Moreover, H&E-stained tissue section images of major organs showed no remarkable organ damage in all groups (Figure 6). *In vivo* safety assessments indicated excellent biocompatibility for lip(PFTBA + PTX) with or without RT. Together, these results demonstrate synergistic liposomes with good biocompatibility could enhance RT efficacy to obtain better tumor inhibition compared to any single treatment.

## Conclusions

In this study, pleiotropic liposomes containing PFTBA and PTX have been prepared using a simple, one-step ultrasonication method, and were found to be promising RT sensitizers. These pleiotropic liposomes showed retained and efficient local accumulation in tumors. PFTBA in liposomes significantly improved tumor hypoxia and PTX in liposomes arrested the tumor cell cycle, and this effect was more pronounced when combined with PFTBA. When combined with RT, lip(PFTBA + PTX) contributed the most effective synergistic tumor growth inhibition compared to the respective mono-therapies. This work reveals that pleiotropic liposomes are safe and biocompatible, and that they are effective at tumor targeting, retention and inhibition when combined with RT. It is hoped that this work will eventually translate into clinical applications for cancer treatment.

## Supplementary Material

IDRD_Liu_et_al_Supplemental_Content.docx
