# Supplementary material for: Enhancement of radiotherapy efficacy by pleiotropic liposomes encapsulated paclitaxel and perfluorotributylamine
Source: Drug Deliv. 2017 Sep 22;24(1):1419–28. doi: 10.1080/10717544.2017.1378939 (PMC8241066; doi:10.1080/10717544.2017.1378939)
Supplement: IDRD_Liu_et_al_Supplemental_Content.docx [file IDRD_A_1378939_SM5297.docx]

**Enhancement of radiotherapy efficacy by pleiotropic liposomes encapsulated paclitaxel and perfluorotributylamine**


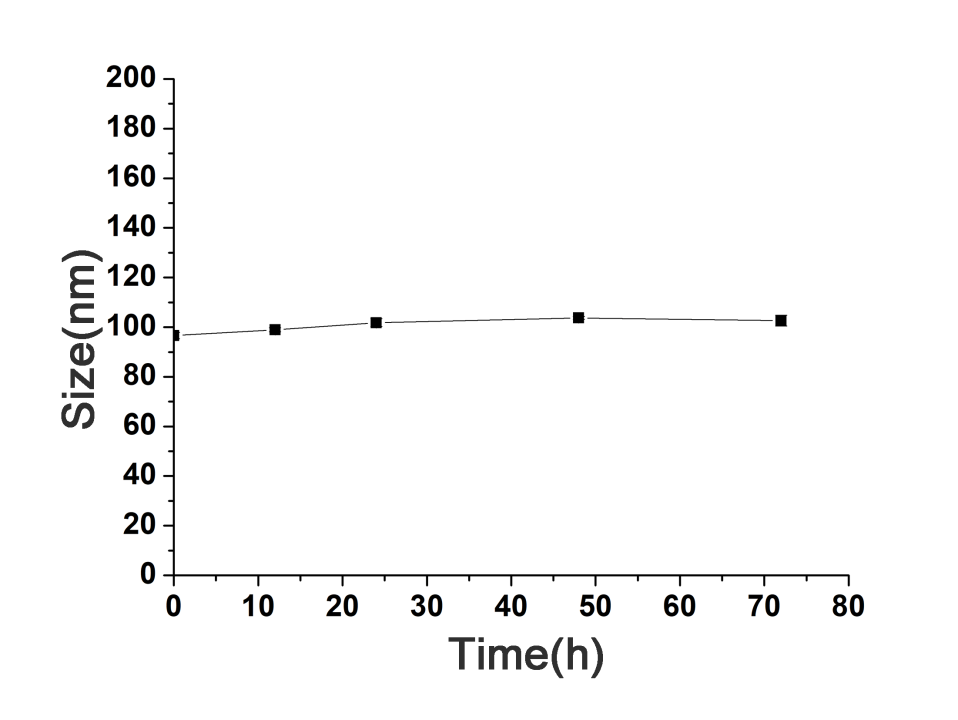


**Figure S1** Stability of lip(PFTBA+PTX) at 25°C for 72 h.


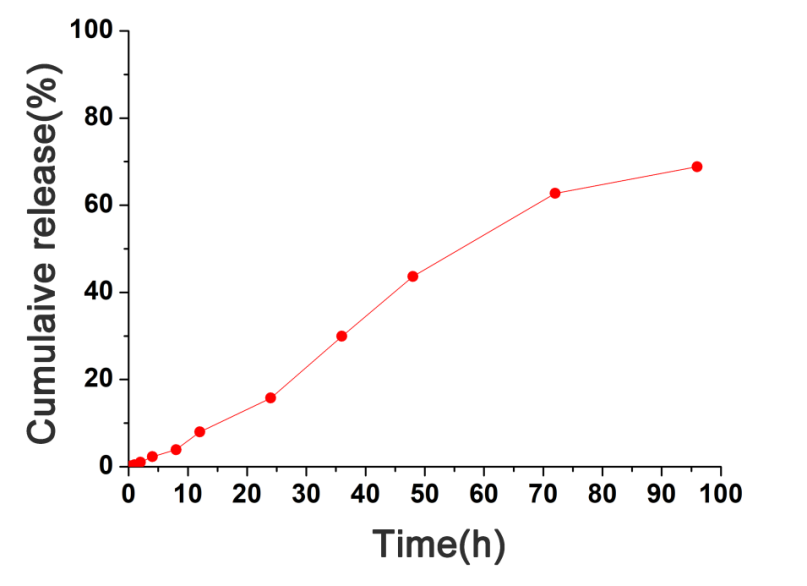


**Figure S2** PTX-release behavior from lip(PFTBA+PTX) in release media (PBS containing 2% fetal bovine serum) at 37°C for 96 h.
